# Toward a Holistic Communication Approach to an Automated Vehicle's Communication With Pedestrians: Combining Vehicle Kinematics With External Human-Machine Interfaces for Differently Sized Automated Vehicles

**DOI:** 10.3389/fpsyg.2022.882394

**Published:** 2022-07-28

**Authors:** Merle Lau, Meike Jipp, Michael Oehl

**Affiliations:** ^1^Institute of Transportation Systems, German Aerospace Center (DLR), Braunschweig, Germany; ^2^Institute of Transport Research, German Aerospace Center (DLR), Berlin, Germany

**Keywords:** automated vehicles, vehicle size, pedestrians, external human-machine interface (eHMI), vehicle kinematics

## Abstract

Future automated vehicles (AVs) of different sizes will share the same space with other road users, e. g., pedestrians. For a safe interaction, successful communication needs to be ensured, in particular, with vulnerable road users, such as pedestrians. Two possible communication means exist for AVs: vehicle kinematics for implicit communication and external human-machine interfaces (eHMIs) for explicit communication. However, the exact interplay is not sufficiently studied yet for pedestrians' interactions with AVs. Additionally, very few other studies focused on the interplay of vehicle kinematics and eHMI for pedestrians' interaction with differently sized AVs, although the precise coordination is decisive to support the communication with pedestrians. Therefore, this study focused on how the interplay of vehicle kinematics and eHMI affects pedestrians' willingness to cross, trust and perceived safety for the interaction with two differently sized AVs (smaller AV vs. larger AV). In this experimental online study (*N* = 149), the participants interacted with the AVs in a shared space. Both AVs were equipped with a 360° LED light-band eHMI attached to the outer vehicle body. Three eHMI statuses (no eHMI, static eHMI, and dynamic eHMI) were displayed. The vehicle kinematics were varied at two levels (non-yielding vs. yielding). Moreover, “non-matching” conditions were included for both AVs in which the dynamic eHMI falsely communicated a yielding intent although the vehicle did not yield. Overall, results showed that pedestrians' willingness to cross was significantly higher for the smaller AV compared to the larger AV. Regarding the interplay of vehicle kinematics and eHMI, results indicated that a dynamic eHMI increased pedestrians' perceived safety when the vehicle yielded. When the vehicle did not yield, pedestrians' perceived safety still increased for the dynamic eHMI compared to the static eHMI and no eHMI. The findings of this study demonstrated possible negative effects of eHMIs when they did not match the vehicle kinematics. Further implications for a holistic communication strategy for differently sized AVs will be discussed.

## Introduction

Participation in today's road traffic requires mutual consideration among all traffic participants (TPs) (German Road Traffic Regulations StVO, 2013; Färber, 2016). In particular, pedestrians are highly dependent on mutual consideration and communication with other TPs as traffic accidents with pedestrians have the highest risk of causing serious injury of any type of road accident (World Health Organisation, 2013). This risk is even higher for pedestrians when they interact with larger vehicles (Tyndall, 2021). Therefore, communication is overall highly relevant to clarifying misunderstandings which can have fatal consequences (Färber, 2016; Rasouli et al., 2017).

In today's traffic, pedestrians communicate implicitly and explicitly with other TPs (Rasouli et al., 2017). Pedestrians typically use implicit communication signals, i.e., driving behavior, to anticipate the vehicle's actions and to plan their behavior accordingly (Dey and Terken, 2017; Ezzati Amini et al., 2019; Lee et al., 2020). However, informal explicit communication signals between vehicles and pedestrians become highly relevant in short distances and in low-speed scenarios, e.g., via eye contact (Färber, 2016; Dey and Terken, 2017; Lee et al., 2020). Explicit communication signals are perceived as supportive to clarify misunderstandings before they can cause accidents (Merat et al., 2018; Stanciu et al., 2018; Schieben et al., 2019a). Overall, both, implicit and explicit, communication signals make it possible to communicate in today's traffic. Nonetheless, the question arises to what extent the interplay of implicit and explicit communication will influence pedestrians' interaction with AVs.

A change toward a mixed traffic environment, including AVs, manually-driven vehicles, and other traffic participants (TPs), is going to happen in the foreseeable future. This mixed traffic will require adequate communication between all TPs to ensure safety, efficiency, and acceptance (Habibovic et al., 2018; Schieben et al., 2019a; Dey et al., 2020b). Implicit and explicit communication means for AVs have been under investigation and results showed that both communication means have the potential to enhance pedestrians' communication with AVs in future mixed traffic (Lee et al., 2019; Bengler et al., 2020; Dey et al., 2020a; Rettenmaier et al., 2020; Schieben et al., 2020; Rettenmaier and Bengler, 2021). However, in most studies either the implicit communication or the explicit communication was varied and the interplay of both was not considered sufficiently yet, in particular, for differently sized AVs. Therefore, this study aims to investigate the interplay of both communication means for pedestrians' interaction with two differently sized AVs in a shared space as an example of a low-speed and low-distance traffic scenario.

### Role of Implicit Communication

Implicit communication signals are sent directly to the traffic environments, however, the perception and further interpretation within the relevant context are needed to understand the signals' message (Färber, 2016; Risto et al., 2017; Bengler et al., 2020; Markkula et al., 2020; Schieben et al., 2020). Current studies indicate that pedestrians primarily use implicit communication to cooperate with other TPs (Risto et al., 2017; Bengler et al., 2020) and base their crossing decision mostly on implicit signals (Beggiato et al., 2017; Dey and Terken, 2017; Lee et al., 2020).

Focusing on future urban traffic, implicit communication remains a highly relevant indicator for pedestrians' crossing decisions, e.g., the vehicle kinematics (Rasouli et al., 2017; Ackermann et al., 2019a,b; Dietrich et al., 2020). The vehicle kinematics can serve as a communication mean for AVs to transmit implicit information, including lateral or longitudinal motions, to the surrounding traffic environment (Risto et al., 2017; Ackermann et al., 2019b; Bengler et al., 2020; Rettenmaier and Bengler, 2021). For example, the initiated vehicle's deceleration at a crossing could be interpreted by pedestrians as a sign that the vehicle gives way (Bengler et al., 2020). Dietrich et al. (2020) investigated the effect of different deceleration rates and pitch angles on pedestrians' interaction with AVs. The results showed that pedestrians initiated their crossing significantly earlier when the AV showed a defensive deceleration. The relevance of the deceleration for the interaction with pedestrians was also demonstrated by Ackermann et al. (2019b), i.e., shorter reaction times by pedestrians to indicate the vehicle's deceleration with higher deceleration rates. Overall, implicit communication, i.e., vehicle kinematics, is a highly relevant indicator of pedestrians' crossing behavior (Ackermann et al., 2019b; Dey et al., 2020a).

### Role of Explicit Communication

Explicit communication signals transmit direct information to the surrounding traffic environment, e.g., via eye contact or hand gesture (Färber, 2016; Markkula et al., 2020; Schieben et al., 2020). Recent studies showed that explicit communication signals could serve as an additional safety check-in low-speed and low-distance traffic situations to clarify misunderstandings in uncertain and ambiguous traffic situations (Dey and Terken, 2017; Sucha et al., 2017; Kitazaki and Daimon, 2018; Lee et al., 2020). In future mixed traffic, pedestrians will no longer be able to communicate explicitly with AVs as they are used to due to the absence of a human driver (Merat et al., 2018; Faas et al., 2020; Schieben et al., 2020; Li et al., 2021).

To enable the explicit communication with AVs, an external human-machine interface (eHMI) positioned on the outside of the vehicle transmits explicit communication signals to the surrounding traffic environment, e.g., about the vehicle's automation status (VAS) or the vehicle's intention (Schieben et al., 2019a; Bengler et al., 2020; Dey et al., 2020b). External HMIs are beneficial to solve ambiguities and clarify misunderstandings in low-speed and low-distance, e.g., in unsignalized and signalized traffic situations (World Health Organisation, 2013; Merat et al., 2018; Schieben et al., 2019b; Faas et al., 2020, 2021; Kaleefathullah et al., 2020; Lee et al., 2021; Wilbrink et al., 2021). Light-based eHMIs present a promising solution to transmit explicit information (Mahadevan et al., 2018; Schieben et al., 2019a; Dey et al., 2020a; Faas et al., 2020). Moreover, light-based eHMIs could present different levels of information richness, e.g., the VAS, the vehicle's intention, or the vehicle's perception (Schieben et al., 2019a; Faas et al., 2020; Lau et al., 2021a; Wilbrink et al., 2021). Previous research showed that pedestrians preferred a dynamic eHMI that presented explicit information about the vehicle's intention plus the VAS and were not satisfied with the mere static presentation of the automation status (VAS) (Faas et al., 2020; Lau et al., 2021a; Wilbrink et al., 2021).

Regarding the effects of eHMIs, pedestrians perceived an AV with eHMI as generally more trust-worthy (Kaleefathullah et al., 2020) and felt safer in interactions with eHMI compared to no eHMI (Kettwich et al., 2019; Schieben et al., 2019a,b). Focusing on pedestrians' willingness to cross, contrasting results exist for pedestrians' interaction with AVs. On the one hand, studies clearly showed that pedestrians were more willing to cross when the interacting AV communicated via eHMI compared to no eHMI (Böckle et al., 2017; Lundgren et al., 2017; Deb et al., 2018; Habibovic et al., 2018; Clercq et al., 2019; Dey et al., 2019, 2022; Ackermans et al., 2020). On the other hand, Clamann et al. (2017) conducted a field study and did not find any effect of an eHMI on pedestrians' willingness to cross compared to no eHMI. However, the participants in this study stated that an eHMI is beneficial for their interaction with an AV (Clamann et al., 2017).

### Joint Role of Implicit and Explicit Communication

The combination of both communication means could support the future interaction with AVs toward a holistic communication approach when both means are well-coordinated (Dey et al., 2020a,b; Dietrich et al., 2020). In a realistic vehicle study by Dey et al. (2020a), pedestrians interacted with an automated car that showed different motion patterns regarding the vehicle kinematics in combination with a light-based eHMI on an unsignalized crossing. Results indicated that gentle braking with a deceleration rate of 2.4 m/s^2^ which started at a distance of 45 m away from the pedestrian and stopped at a 5-m distance could contribute to the overall traffic safety in combination with an eHMI showing the vehicle's intention (Dey et al., 2020a). If the vehicle kinematics contradicted the message of the eHMI, pedestrians primarily based their willingness to cross on the vehicle kinematics rather than the eHMI communication (Dey et al., 2020a). In contrast, a study by Kaleefathullah et al. (2020) revealed that when the eHMI was on, but the AV did not indicate a braking process, pedestrians still crossed the street. This result demonstrated possible negative effects, i.e., over-trust, which have been also found by other studies (Kitazaki and Daimon, 2018; Holländer et al., 2019; Lee et al., 2021). Lee et al. (2021) investigated the effect of text-based eHMIs and showed that the participants behaved less carefully when interacting with an AV equipped with eHMI. As an explanation, the authors described an over-trust in the communication abilities of the AV (Lee et al., 2021). Overall, such negative effects would come at high risk for pedestrians due to their vulnerability and, thus, need further investigation (Färber, 2016; Rasouli et al., 2017).

A possible explanation for the occurrence of negative effects of eHMI could be that humans do not always interpret the communication signals correctly (Smeets et al., 1996; DeLucia, 2008; Ackermann et al., 2019b; Lee et al., 2022). According to the Elaboration Likelihood Model (Petty and Cacioppo, 1986), humans can elaborate communication signals by two routes: the central and the peripheral route. The central route describes the careful consideration of all the presented information. The peripheral route describes the consideration of simple salient cues which are signals that attract human perception and direct human attention, e.g., light or acoustic signals (Petty and Cacioppo, 1986; Wickens, 2021). Regarding pedestrians' interaction with AVs, one would assume that pedestrians carefully consider what AVs communicate implicitly and explicitly (central route), in particular, as miscommunication comes with a high risk to get injured (Ackermann et al., 2019a). However, studies that demonstrated the negative effects of eHMIs manifested that humans do not always focus on the correct information but rather direct their attention to the explicit signals that the eHMI presented (peripheral route). This might be due to the fact that urban traffic presents a complex environment in which pedestrians need to make fast decisions under the influence of various factors, e.g., the interaction with other TPs. This in turn could lead to mistakes (Rasouli and Tsotsos, 2020; Wickens, 2021). Therefore, it becomes highly relevant to investigate how pedestrians elaborate the presented implicit and explicit communication signals, i.e., vehicle kinematics and eHMIs, to define an AV's holistic communication strategy that does not endanger pedestrians in future urban traffic.

All in all, both means of communication, i.e., vehicle kinematics and eHMI, have the potential to support pedestrians' interaction with AVs in future urban traffic. However, the combination of both means and their precise coordination needs further clarification for AVs' communication with pedestrians toward a holistic communication strategy. Additionally, it needs to be addressed to what extent the vehicle size will affect pedestrians subjectively, i.e., pedestrians' willingness to cross, trust, and perceived safety as current research on the interplay of vehicle kinematics and eHMI does not address the effect of vehicle size for the interaction with pedestrians (Dey et al., 2020b).

### Role of Vehicle Size

The vehicle size can influence pedestrians' interaction with differently sized vehicles in urban traffic (Caird and Hancock, 1994; DeLucia, 2008, 2013; Petzoldt, 2016). This has been investigated by focusing on objective and subjective measurements (Horswill et al., 2005; DeLucia, 2013; Petzoldt, 2016; Beggiato et al., 2017; Levulis et al., 2018). Regarding objective measurements, results showed that humans perceived larger vehicles to arrive earlier compared to smaller vehicles (Petzoldt, 2016; Beggiato et al., 2017; Petzoldt et al., 2017). These findings stood in line with the size-arrival effect which describes that large objects are perceived to arrive earlier than small objects although they had the same arrival time (DeLucia, 2008, 2013). Moreover, pedestrians selected larger time gaps for a larger vehicle compared to smaller vehicles, i.e., showed a more conservative crossing behavior (Petzoldt et al., 2017; Hensch et al., 2021). As a possible explanation, Petzoldt et al. (2017) pointed out that the perceived risk of an accident and pedestrians' individual state or traits could influence the expected time-of-arrival (TTA) and their gap acceptance. Regarding subjective measurements, pedestrians also evaluated larger vehicles as more threatening and stronger compared to smaller ones (Petzoldt, 2016; Dey et al., 2017). Overall, previous research manifested an effect of vehicle size on pedestrians' subjective evaluation and their actual crossing behavior. There is a clear connection between pedestrians' subjective evaluation and their actual decision to cross the street (Ezzati Amini et al., 2019). Therefore, it is highly relevant to address the question if differently sized AVs could also affect pedestrians' willingness to cross, trust, or perceived safety.

### Research Aim and Hypotheses

This study aims to investigate pedestrians' interaction with two differently sized AVs (smaller AV vs. larger AV) focusing on the interplay of eHMI status (no eHMI vs. static eHMI vs. dynamic eHMI) and vehicle kinematics (yielding vs. non-yielding) on a shared space. Very preliminary results of this study have been already published (Lau et al., 2021b).

Overall, this study investigated the effects of vehicle size, vehicle kinematics, and eHMI status individually as well as the interplay of vehicle kinematics and eHMI status on pedestrians' willingness to cross, trust and perceived safety. Based on the previously given theoretical background, the following is hypothesized:

Hypothesis 1 (H1): *Pedestrians' willingness to cross, trust and perceived safety* is higher for a smaller AV compared to a larger AV.

Hypothesis 2 (H2): *Pedestrians' willingness to cross, trust and perceived safety* is higher for both vehicle sizes when the AV yields compared to when it does not yield.

Hypothesis 3 (H3): *Pedestrians' willingness to cross, trust and perceived safety* is higher for both vehicle sizes when the AV is equipped with a dynamic eHMI compared to a static eHMI or no eHMI at all.

Hypothesis 4 (H4): The effect of vehicle kinematics on *pedestrians' willingness to cross, trust and perceived safety* differs depending on the eHMI status for the interaction with both vehicle sizes.

Hypothesis 5 (H5): When the AV does not yield, *pedestrians' willingness to cross, trust and perceived safety* will be based on the vehicle kinematics and not the eHMI communication for both vehicle sizes.

## Methods

This experimental study used an online-based methodological approach to investigate pedestrians' interaction with two differently sized AVs (smaller vs. larger AV) in an urban environment. Both AVs were equipped with an LED light-band eHMI and displayed different eHMI statuses (no eHMI vs. static eHMI vs. dynamic eHMI). Moreover, the vehicle kinematics were varied for both AVs (yielding vs. non-yielding).

### Participants

This study was conducted with 149 participants (48 women) aged between 19 and 71 years (*M* = 35.41; *SD* = 12.68). To evaluate the extent to which the participants use technology, the participants completed the affinity for technology interaction (ATI) questionnaire which consists of nine items (Franke et al., 2018). The participants indicated a mid-ranged ATI with *M* = 4.38 (*SD* = 0.90) on a 6-point scale (from 1 = “completely disagree” to 6 = “completely agree”) (Franke et al., 2018). To assess the participants' familiarity with the experimental setting, it was questioned how and where they carry their errands on a regular basis. Of all participants, 92 participants stated that they frequently run errands on foot. Moreover, 123 participants reported that they move primarily in urban areas and only 26 participants stated that they move primarily in rural areas. All participants have heard of AVs (*N* = 149) and were interested in AVs (*M* = 3.93, *SD* = 1.08; from 1 = “completely disagree” to 5 = “completely agree”). In accordance with the Declaration of Helsinki, informed consent was obtained from all participants before the experiment. The participants were recruited from social networks and from an internal database. During the experiment, the participants were allowed to stop the study at any point without justification or consequence. As an expense, the participants could participate in a raffle of four online vouchers in the amount of 25 euros on a voluntary basis. For their participation in the raffle, they could enter their email address which was saved separately from the experimental data to ensure anonymity.

As this study was conducted online, a great emphasis was placed on the video functionality, validity, and diligence of the participants' ratings. Before the experimental phase, the video functionality was tested with a test video. All participants (*N* = 149) indicated that they were able to play the test video properly. After the experimental phase, further questions on participants' perception of the light-band and the vehicle kinematics for both vehicles separately were asked. Overall, 29 participants answered that they were unsure or did not perceive changes in the vehicle kinematics and, therefore, were excluded from further analysis. Moreover, 4 participants denied that they could see the light-band well and were also excluded. Additionally, it was asked how carefully they conducted the questionnaire on a 5-point Likert scale (from 1 = “very careless” to 5 = “very careful”). All participants answered with rather careful (*N* = 26), careful (*N* = 83) and very careful (*N* = 40).

### Study Design

This study was conducted as a 2 × 2 × 3 research design with vehicle size (smaller AV, larger AV), vehicle kinematics (yielding, non-yielding), and eHMI status (no eHMI, static eHMI, and dynamic eHMI) manipulated within the participants. This research design consisted of a non-matching condition for each vehicle size in which the dynamic eHMI falsely indicated that the vehicle yields, although no yielding behavior was shown by the vehicle kinematics.

### Independent Variables

#### Vehicle Size

**Figure 1:**
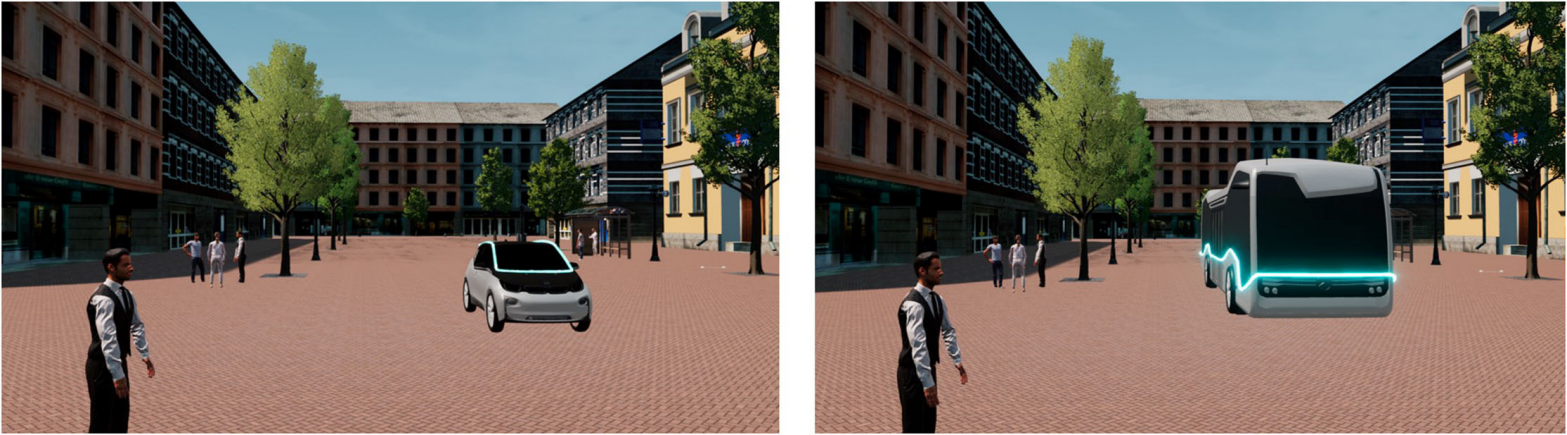
Experimental setting of this study: The smaller AV (left) with static eHMI and the larger AV (right) with pulsating dynamic eHMI approached the pedestrian from the left-hand side on the shared space.

The videos showed two differently sized vehicles. Based on Schieben (2020), the smaller AV was related to a BMW model i3 which was also investigated in other studies (e.g., Weber et al., 2019; Wilbrink et al., 2021). The larger AV was related to a Mercedes Benz future public bus. Both vehicles presented the same eHMI communication strategies (Figure 1).

#### Vehicle Kinematics

**Figure 2:**
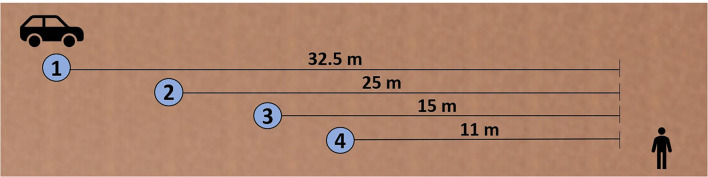
Procedure for the yielding conditions in this study: 1. Video starts in 32.5 m, 2. First deceleration from 30 to 20 km/h starting in 25 m, 3. Second deceleration from 20 km/h to 2 km/h starting in 15 m, 4. Video stop in 11 m (distances measured from the vehicle's bumper to the pedestrians' position; m = meter).

The vehicle kinematics were varied at two levels, yielding and non-yielding. For the yielding conditions, the overall procedure consisted of four steps (Figure 2). The video started when the AV was at a distance of 32.5 m from the pedestrian (Step 1). After this, the AV performed a two-step deceleration. The first deceleration (30–20 km/h) started at a 25 m distance to the pedestrian and was performed with an average deceleration rate of −1.92 m/s^2^ over 10 m (Step 2). The second deceleration (20 to 2 km/h) started at a 15 m distance to the pedestrian and was performed with an average deceleration of −3.83 m/s^2^ within 4 m (Step 3). The video stopped at a predefined distance of 11 m (Step 4). At this point, the vehicle still had a speed of 2 km/h. Overall, the deceleration of the AVs was set with the goal to create a traffic situation with high uncertainty without provoking a conflict. For the non-yielding conditions, the video also started at a distance of 32.5 m to the pedestrian. After this, the vehicle drove at a constant speed of 30 km/h toward the pedestrian. The video stopped at the predefined distance of 11 m. Distances were measured from the vehicle's bumper to the pedestrians' position.

#### eHMI Status

Both vehicle sizes (smaller AV and larger AV) were equipped with a LED light-band eHMI positioned under the vehicle's windshield that presented different eHMI communication strategies. The static eHMI showed a continuously enlightened LED light-band eHMI from the beginning of the video. This indicated the vehicle's automation status (VAS). The dynamic eHMI showed the vehicle's yielding intention on top of the VAS. In conditions with dynamic eHMI, the LED light-band eHMI was continuously enlightened from the start of the video and started to pulsate at 25 m (distance measured from the vehicle's bumper to the pedestrians' position) at a frequency rate of 0.66 Hz. The distance of 25 m was chosen to ensure no advantage of the communication via eHMI over the vehicle kinematics or the other way around. The experimental condition “no eHMI” was without LED light-band eHMI and served as a baseline.

### Dependent Variables

After each video presentation, the participants evaluated their willingness to cross, trust, and perceived safety. Pedestrians' willingness to cross was measured with the question “What is your willingness to cross in front of the vehicle at the end of the video?” on a 7-point Likert scale (from 1 = “very low” to 7 = “very high”). Participants' trust (“How much would you trust the vehicle to stop for you?”) and perceived safety (“For my personal safety, I found the behavior of the vehicle to be safety-enhancing.”) was assessed on a 7-point Likert scale from 1 = “disagree” to 7 = “agree.” To get a deeper insight into the effects of vehicle kinematics and eHMI on pedestrians' willingness to cross, two additional subjective measurements were included, i.e., the perceived support of the vehicle kinematics (“The vehicle behavior has helped me to assess my willingness to cross in front of the vehicle.”) and the perceived support of the eHMI (“The light band has helped me to assess my willingness to cross in front of the vehicle.”). Both items were evaluated on a 7-point Likert scale (from 1 = “disagree” to 7 = “agree”). The perceived support of the vehicle kinematics was evaluated after each video presentation and the perceived support of the eHMI was only evaluated for conditions in which the eHMI presented information (static eHMI, dynamic eHMI).

### Procedure

The online experiment started when the participants clicked on the website link. The conduction and data recording took place with the SoSci questionnaire software (Leiner, 2019). In the beginning, the participants were informed about the conditions of participation, and they gave their consent to participate. Moreover, they were instructed the following: Firstly, to conduct the questionnaire on a computer and not a tablet or smartphone, secondly, to dim light sources in their environment, and, thirdly, to play the following videos in full-screen mode. On the first page of the questionnaire, the participants were asked to fill out the demographic questionnaire and ATI questionnaire (see Section Participants). Before the experimental phase started, the participants were informed step-by-step about the experimental setting, the two AVs, and the eHMI communication strategies with short tutorial videos and additional written instructions. This step was done to give the participants detailed information about the experiment and to let them familiarize themselves with the online environment.

In the experimental phase, each of the participants saw twelve video sequences which were shown from an egocentric perspective (Table 1). The video length for the yielding conditions was 9 s and for the non-yielding conditions 7 s. The traffic environment was designed using the software Unreal Engine (Version 4.24.2.). The traffic scenario was a shared space and the same for all conditions (Figure 1). Shared space was chosen for investigation as in this low-speed and low-distance scenario the right of way is not clarified yet and not explicitly defined by the traffic signs in this case and, thus, misunderstandings could occur. Previous research has shown that explicit communication is highly relevant when misunderstandings occur (Dey et al., 2017; Lee et al., 2019). All twelve experimental conditions were presented in randomized order to prevent any learning effects (Table 1). At the beginning of each video, the participants stood in the same position and looked to the left from where the smaller and larger AV drove toward them. All videos stopped at a predefined distance of 11 m. The stopping point should represent the point of high uncertainty without frightening the participants and was set based on previous internal evaluations. At the end of the experiment, the participants were asked to rate their interaction with both AVs separately (smaller AV vs. larger AV) regarding a set of eight adjectives (threatening, large, pleasant, dangerous, strong, familiar, safe, close) on a 7-Likert scale (from 1 = “disagree” to 7 = “agree”) which was based on by Petzoldt (2016). The whole online experiment took ~25 min.

**Table 1:** Overview of this study's experimental conditions presented in the short video sequences (presented in randomized order).

**Experimental**	**Vehicle**	**Vehicle**	**eHMI**
**conditions**	**size**	**kinematics**	**status**
1	Smaller AV	Yielding	No eHMI
2			Static eHMI
3			Dynamic eHMI
4	Smaller AV	Non-yielding	No eHMI
5			Static eHMI
6			Dynamic eHMI
7	Larger AV	Yielding	No eHMI
8			Static eHMI
9			Dynamic eHMI
10	Larger AV	Non-yielding	No eHMI
11			Static eHMI
12			Dynamic eHMI

## Results

### Statistical Approach

The data analysis started with a data validation check to evaluate the participants' assessment of the vehicle's characteristics with both AVs (smaller AV vs. larger AV) in the videos. For the *t*-tests, Cohen's *d*_*z*_ was used and interpreted as effect size [*d*_*z*_ = 0.2 (small effect), *d*_*z*_ = 0.5 (medium effect) and *d*_*z*_ = 0.8 (large effect)] (Cohen, 1988). Furthermore, we used a 2 × 2 × 3 repeated-measures ANOVA to investigate the effect of vehicle size, vehicle kinematics, and eHMI status (all within-participants) on pedestrians' willingness to cross, trust, and perceived safety. The assumption of normally distributed data was given due to the sample size (Field, 2009). Sphericity was calculated with Mauchly's W test and Huynh-Feldt corrections were applied when the assumption of sphericity was violated (Field, 2009). Partial eta-squared ($\eta_{\text{p}}^{2}$) was used as effect size and for interpretation: $\eta_{\text{p}}^{2}$ ≤ 0.01 (small effect), $\eta_{\text{p}}^{2}$ ≤ 0.06 (medium effect) and $\eta_{\text{p}}^{2}$ < 0.14 (large effect) (Cohen, 1988). For post-hoc tests, all additional *t*-tests were conducted with a Bonferroni-corrected *p*-value *(p* < 0.003). In additional comparisons, the effect of eHMI status (no eHMI vs. static eHMI vs. dynamic eHMI) was compared for each AV (smaller and larger AV) for the non-yielding conditions. This was done to investigate the conditions in which the displayed eHMI information was consistent with the vehicle kinematics (no eHMI, static eHMI) or inconsistent (dynamic eHMI) for both differently sized AVs individually. To get a deeper insight into the effects of vehicle kinematics and eHMI on pedestrians' willingness to cross, the relationship between pedestrians' willingness to cross, the perceived support of the vehicle kinematics, and the perceived support of the eHMI was investigated for both AVs with Pearson's *r* (*N* = 149). According to Cohen (1988), Pearson's *r* correlations were interpreted as followed: *r* <0.3 (small effect), *r* = 0.3–0.5 (medium effect) and *r* > 0.5 (large effect).

### Data Validation Check

**Figure 3:**
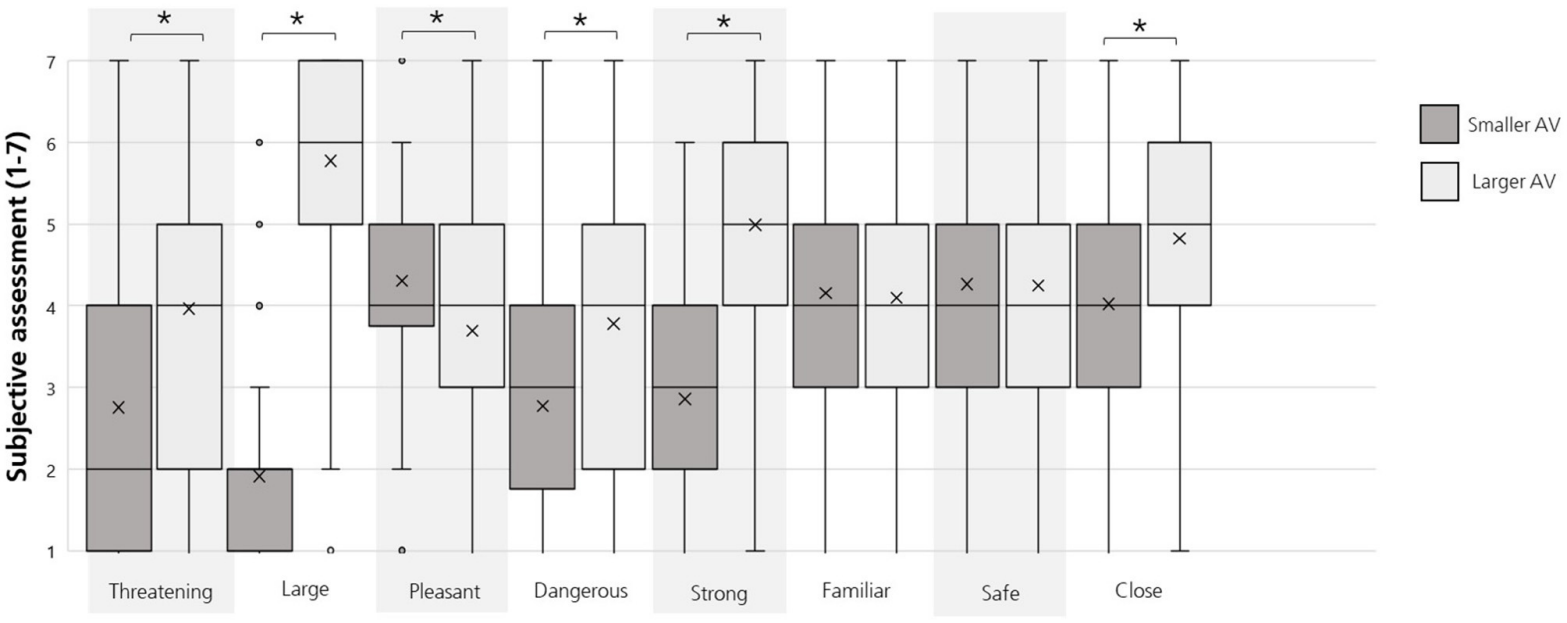
Boxplots for participants' subjective assessments on each vehicle's (smaller AV vs. larger AV) characteristics for their interactions in the videos. *Note*. Crosses = means; lines = medians. Bonferroni-corrected *p-*value * < 0.003.

As this experimental study was conducted online, we wanted to check to what extent the demonstrated AVs in the videos led to a similar subjective assessment on the vehicle's characteristics as described in previous studies (Petzoldt, 2016; Dey et al., 2017). The statistical results are displayed in Table 2 and boxplots in Figure 3.

**Table 2:** *T*-test results comparing participants' assessment of vehicles' characteristics (smaller vs. larger vehicle).

	**Smaller AV**		**Larger AV**					
	***M***	***SD***	***M***	***SD***	***df***	***t***	***p***	***d_***z***_***
Threatening	2.77	1.77	3.99	1.85	148	−7.29	0.001^**^	0.59
Large	1.93	1.10	5.81	1.24	148	−29.03	0.001^**^	1.00
Pleasant	4.34	1.51	3.72	1.31	148	4.42	0.001^**^	0.36
Dangerous	2.79	1.51	3.81	1.64	148	−7.33	0.001^**^	0.59
Strong	2.88	1.35	5.03	1.49	148	−14.99	0.001^**^	1.23
Familiar	4.18	1.59	4.13	1.55	148	0.43	0.67	0.03
Safe	4.30	1.45	4.28	1.40	148	0.18	0.86	0.02
Close	4.05	1.49	4.86	1.25	148	−6.42	0.001^**^	0.53

** *p < 0.001*.

The results showed that a larger AV was perceived as significantly more threatening, larger, less pleasant, more dangerous, stronger, and closer compared to the smaller AV in this study (Figure 3). Thus, it can be assumed that the vehicles presented in the videos were evaluated differently which stands in line with previous studies (Petzoldt, 2016; Dey et al., 2017).

### Willingness to Cross

**Figure 4:**
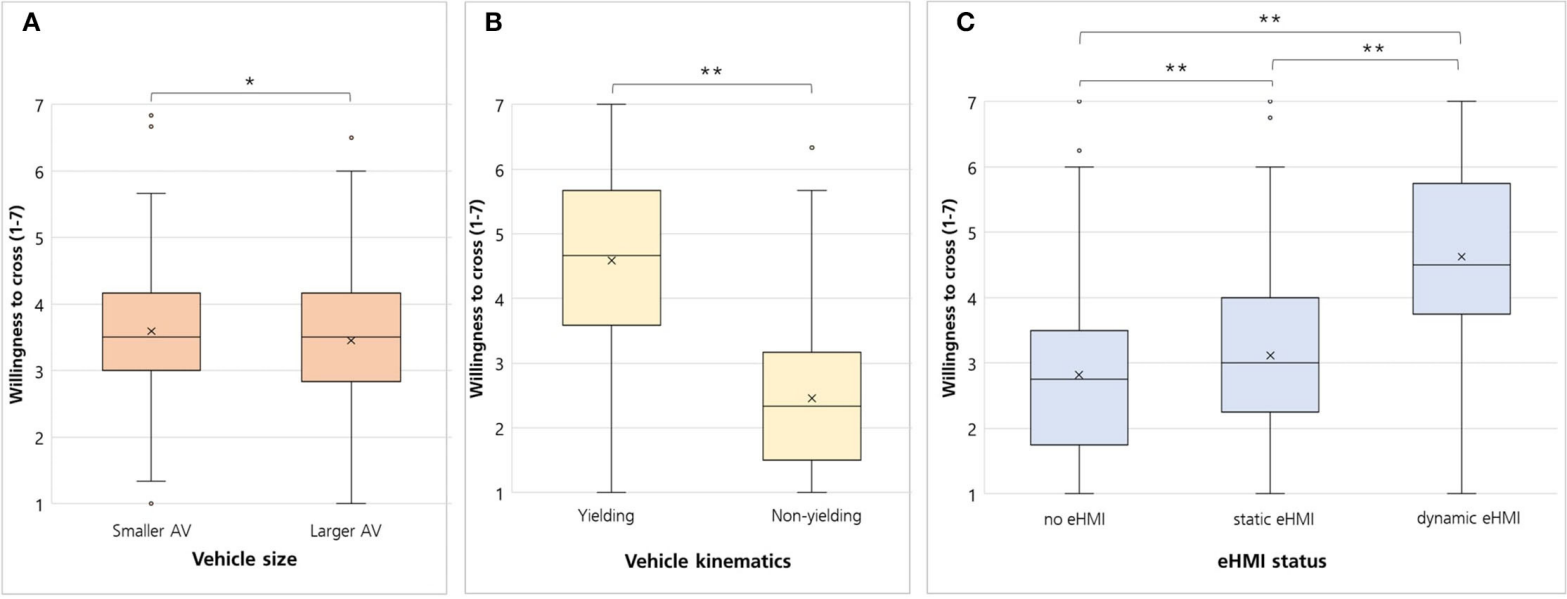
Boxplots for pedestrians' willingness to cross focusing on the variables **(A)** vehicle size (smaller vs. larger AV), **(B)** vehicle kinematics (yielding vs. non-yielding), and **(C)** eHMI status (no eHMI vs. static eHMI vs. dynamic eHMI). Crosses = means; lines = medians; **p < 0.001 *p < 0.01.

The inferential statistical analysis showed a significant main effect for vehicle size [*F*_(1,148)_ = 6.69, *p* = 0.006, $\eta_{\text{p}}^{2}$ = 0.043], however, with a rather small effect size (Figure 4). The willingness to cross was higher for the smaller AV (*M* = 3.59, *SD* = 1.07) compared to the larger AV (*M* = 3.45, *SD* = 1.07; *p* < 0.01) (Figure 4). Additionally, a significant main effect was found for vehicle kinematics [*F*_(1,148)_ = 255.67, *p* < 0.001, $\eta_{\text{p}}^{2}$ = 0.633] indicating that the yielding vehicle led to a higher willingness to cross (*M* = 4.59, *SD* = 1.41) compared to the non-yielding vehicle (*M* = 2.46, *SD* = 1.17; *p* < 0.001) (Figure 4). Furthermore, the results showed a significant main effect for eHMI status [*F*_(1.48,216.49)_ = 136.09, *p* < 0.001, $\eta_{\text{p}}^{2}$ = 0.479] (Figure 4). Pairwise comparisons with Bonferroni correction revealed that the willingness to cross was higher for the dynamic eHMI (*M* = 4.63, *SD* = 1.42) compared to the static eHMI (*M* = 3.12, *SD* = 1.28; *p* < 0.001) and no eHMI (*M* = 2.82, *SD* = 1.22; *p* < 0.001).

There were no significant interactions for vehicle size^*^vehicle kinematics [*F*_(1,148)_ = 1.29, *p* = 0.26, $\eta_{\text{p}}^{2}$ = 0.009], vehicle size^*^eHMI status [*F*_(1.96,290.13)_ = 1.17, *p* = 0.31, $\eta_{\text{p}}^{2}$ = 0.008], vehicle kinematics^*^eHMI status [*F*_(1.88,278.84)_ = 0.17, *p* = 0.84, $\eta_{\text{p}}^{2}$ = 0.001] and vehicle size^*^vehicle kinematics^*^eHMI status [*F*_(1.99,295.79)_ = 1.82, *p* = 0.16, $\eta_{\text{p}}^{2}$ = 0.012].

#### Pedestrians' Willingness in Relation to Vehicle Kinematics and eHMI

The relationship between pedestrians' willingness to cross (Table 3) and, firstly, pedestrians' perceived support of the vehicle kinematics (Table 4) and, secondly, pedestrians' perceived support of the light-band for their crossing decision (Table 5) was investigated for each experimental condition. This was done to further focus on pedestrians' perceived support of the vehicle kinematics and the eHMI to indicate their willingness to cross.

**Table 3:** Pedestrians' mean willingness to cross regarding the vehicle size (smaller AV, larger AV), vehicle kinematics (yielding, non-yielding), and eHMI status (no eHMI, static eHMI, dynamic eHMI).

	**Smaller AV**			**Larger AV**		
	**No eHMI**	**Static eHMI**	**Dynamic eHMI**	**No eHMI**	**Static eHMI**	**Dynamic eHMI**
Yielding	3.96 (*1.99*)	4.17 (*1.98*)	5.75 (*1.52*)	3.78 (*2.06*)	4.17 (*1.92*)	5.67 (*1.60*)
Non-yielding	1.89 (*1.35*)	2.22 (*1.54*)	3.55 (*2.02*)	1.66 (*1.13*)	1.89 (*1.34*)	3.54 (*2.12*)

*1 = “very low; 7 = “very high”. Mean (Standard deviation [italics]).*

**Table 4:** Perceived support of the vehicle kinematics regarding the vehicle size (smaller AV, larger AV), vehicle kinematics (yielding, non-yielding), and eHMI status (no eHMI, static eHMI, dynamic eHMI).

	**Smaller AV**			**Larger AV**		
	**No eHMI**	**Static eHMI**	**Dynamic eHMI**	**No eHMI**	**Static eHMI**	**Dynamic eHMI**
Yielding	4.51 (*2.04*)	4.76 (*1.89*)	5.68 (*1.42*)	4.51 (*1.99*)	4.72 (*1.86*)	5.62 (*1.57*)
Non-yielding	3.73 (*2.24*)	4.30 (*2.10*)	4.23 (*2.06*)	3.73 (*2.24*)	4.21 (*2.24*)	4.34 (*2.15*)

*1 = “very low; 7 = “very high”. Mean (Standard deviation [italics])*.

**Table 5:** Pedestrians' perceived support of eHMI regarding the vehicle size (smaller AV, larger AV), vehicle kinematics (yielding, non-yielding), and eHMI status (static eHMI, dynamic eHMI).

	**Smaller AV**		**Larger AV**	
	**Static eHMI**	**Dynamic eHMI**	**Static eHMI**	**Dynamic eHMI**
Yielding	2.75 (*1.88*)	5.60 (*1.68*)	2.95 (*1.97*)	5.51 (*1.73*)
Non-yielding	3.46 (*2.20*)	3.97 (*2.23*)	3.48 (*2.26*)	4.21 (*2.25*)

*1 = “very low; 7 = “very high”. Mean (Standard deviation [italics])*.

Firstly, there were high correlations between pedestrians' willingness to cross and the perceived support of the vehicle kinematics for both AVs when the vehicle yielded in combination with no eHMI (smaller AV: *r* = 0.69, *p* < 0.001; larger AV: *r* = 0.68, *p* < 0.001), the static eHMI (smaller AV: *r* = 0.65, *p* < 0.001; larger AV: *r* = 0.7, *p* < 0.001), or the dynamic eHMI (smaller AV: *r* = 0.6, *p* < 0.001; larger AV: *r* = 0.58, *p* < 0.001) with large effect sizes. When the vehicle did not yield, but the dynamic eHMI indicated so, there were significant correlations for both AVs (smaller AV: *r* = 0.24, *p* < 0.001; larger AV: *r* = 0.3, *p* < 0.001) with small and medium effect sizes. No significant correlations were found when the vehicle did not yield in combination with no eHMI or a static eHMI for both AVs (*p* > 0.05).

Secondly, there were significant correlations between pedestrians' willingness to cross and the perceived support of the eHMI for both AVs when the vehicle yielded and was equipped with dynamic eHMI (smaller AV: *r* = 0.53, *p* < 0.001; larger AV: *r* = 0.5, *p* < 0.001). When the vehicle did not yield but the dynamic eHMI indicated so, pedestrians' willingness to cross still highly correlated with the perceived support of the eHMI (smaller AV: *r* = 0.81, *p* < 0.001; larger AV: *r* = 0.73, *p* < 0.001). No correlations were found between pedestrians' willingness to cross and the perceived support of a static eHMI for both AVs (*p* > 0.05).

### Trust

**Figure 5:**
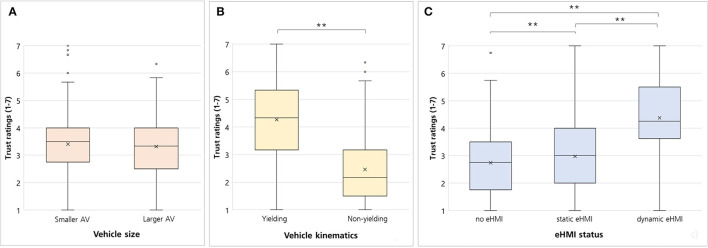
Boxplots for pedestrians' trust focusing on the variables **(A)** vehicle size (smaller AV vs. larger AV), **(B)** vehicle kinematics (yielding vs. non-yielding), and **(C)** eHMI status (no eHMI vs. static eHMI vs. dynamic eHMI). Crosses = means; lines = medians; ^**^p < 0.001.

A significant main effect for vehicle kinematics was found [*F*_(1,148)_ = 212.59, *p* < 0.001, $\eta_{\text{p}}^{2}$ = 0.59; Figure 5]. The participants indicated higher trust ratings when they interacted with the yielding vehicle (*M* = 4.27, *SD* = 1.49) compared to the non-yielding (*M* = 2.46, *SD* = 1.20; *p* < 0.001; Figure 5). Moreover, a significant main effect for eHMI status was found [*F*_(1.53,226.34)_ = 133.85, *p* < 0.001, $\eta_{\text{p}}^{2}$ = 0.475; Figure 5]. Pairwise comparisons with Bonferroni-correction revealed significant differences between all three eHMI statuses, i.e., the dynamic eHMI (*M* = 4.38, *SD* = 1.45) lead to higher trust ratings vs. static eHMI (*M* = 2.97, *SD* = 1.35; *p* < 0.001) vs. no eHMI (*M* = 2.74, *SD* = 1.25; *p* < 0.001). There was no significant main effect for vehicle size [*F*_(1,148)_ = 2.21, *p* = 0.07, $\eta_{\text{p}}^{2}$ = 0.015] and no significant interactions for vehicle size^*^vehicle kinematics [*F*_(1,148)_ = 1.59, *p* = 0.21, $\eta_{\text{p}}^{2}$ = 0.011], vehicle size^*^eHMI status [*F*_(2,296)_ = 0.78, *p* = 0.39, $\eta_{\text{p}}^{2}$ = 0.006], vehicle kinematics^*^eHMI status [*F*_(1.9,281.4)_ = 1.46, *p* = 0.23, $\eta_{\text{p}}^{2}$ = 0.01] and vehicle size^*^vehicle kinematics^*^eHMI status [*F*_(1.97,291.41)_ = 1.62, *p* = 0.20, $\eta_{\text{p}}^{2}$ = 0.011].

### Perceived Safety

**Figure 6:**
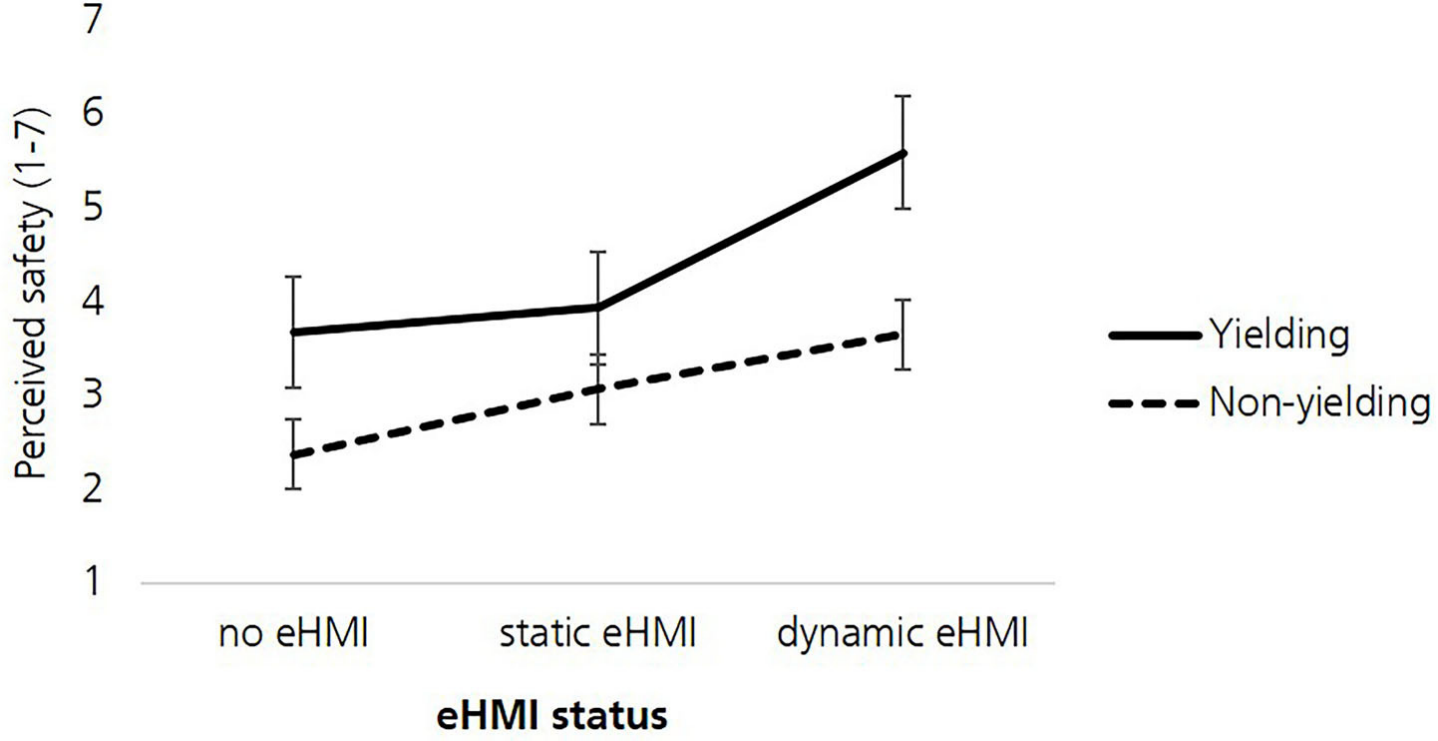
Perceived safety regarding the eHMI status and vehicle kinematics. Error bars: ± 1 *SE*.

The interaction between vehicle kinematics and eHMI status was significant [*F*_(1.59,234.61)_ = 19.33, *p* < 0.001, $\eta_{\text{p}}^{2}$ = 0.116; Figure 6]. The ordinal interaction underlined the interpretability of the two main effects for vehicle kinematics and eHMI status. The main effect for vehicle kinematics was significant [*F*_(1,148)_ = 129.7, *p* < 0.001, $\eta_{\text{p}}^{2}$ = 0.467]. The perceived safety was higher when the vehicle yielded (*M* = 4.40, *SD* = 1.33) vs. not yielded (*M* = 3.03, *SD* = 1.29; *p* < 0.001). Moreover, the main effect for eHMI status was significant [*F*_(1.57,232.96)_ = 120.99, *p* < 0.001, $\eta_{\text{p}}^{2}$ = 0.45]. Pairwise comparisons with Bonferroni correction revealed that the perceived safety was higher for the dynamic eHMI (*M* = 4.62, *SD* = 1.34) vs. static eHMI (*M* = 3.50, *SD* = 1.31; *p* < 0.001) vs. no eHMI (*M* = 3.02, *SD* = 1.3; *p* < 0.001). There was no significant main effect for vehicle size [*F*_(1,148)_ = 0.05, *p* = 0.41, $\eta_{\text{p}}^{2}$ = 0.000] and no significant interactions for vehicle size^*^vehicle kinematics [*F*_(1,148)_ = 0.37, *p* = 0.54, $\eta_{\text{p}}^{2}$ = 0.003], vehicle size^*^eHMI status [*F*_(1.91, 282.5)_ = 0.73, *p* = 0.48, $\eta_{\text{p}}^{2}$ = 0.005] and vehicle size^*^vehicle kinematics^*^eHMI status [*F*_(2,295.37)_ = 2.14, *p* = 0.12, $\eta_{\text{p}}^{2}$ = 0.014].

Post-hoc comparisons revealed that, when the smaller AV did not yield, the participants gave significantly lower ratings of perceived safety when the smaller AV was equipped with dynamic eHMI (*M* = 3.58, *SD* = 1.97) vs. static eHMI (*M* = 3.17, *SD* = 1.79; *t* = 2.29, *p* = 0.023, *n* = 148, *d*_*z*_ = 0.19) and vs. no eHMI *(M* = 2.37, *SD* = 1.62; *t* = 6.73, *p* < 0.001, *n* = 148, *d*_*z*_ = 0.55). When the larger AV did not yield, pedestrians' perceived safety was higher with dynamic eHMI (*M* = 3.73, *SD* = 2.05) vs. static eHMI (*M* = 2.97, *SD* = 1.89; *t* = 3.93, *p* < 0.001, *n* = 148*; d*_*z*_ = 0.32) vs. no eHMI (*M* = 2.37, *SD* = 1.57; *t* = 7.08, *p* < 0.001, *n* = 148, *d*_*z*_ = 0.58).

### Evaluation of Hypotheses

In conclusion, it was hypothesized that pedestrians' willingness to cross, trust, and perceived safety is higher for a smaller AV vs. a larger AV (H1). According to the results, H1 is confirmed only partially, i.e., pedestrians' willingness to cross was higher for a smaller AV vs. larger AV, however, with a rather small effect size. Moreover, it was hypothesized that pedestrians' wiliness to cross, trust and perceived safety is higher when the vehicle yielded compared to when it did not yield for both vehicle sizes (H2). This can be confirmed for all variables. Furthermore, it was hypothesized that pedestrians' willingness to cross, trust, and perceived safety is higher when the AV is equipped with dynamic eHMI vs. static eHMI vs. no eHMI for both vehicle sizes (H3). Overall, this can be confirmed. Additionally, it was hypothesized that the effect of vehicle kinematics differs depending on the eHMI status for all dependent variables for both vehicle sizes (H4). According to the results, H4 was confirmed only for pedestrians' perceived safety. Moreover, it was hypothesized that, when the vehicle did not yield, pedestrians tended to rely on the explicit communication signals rather than the implicit communication signals for both vehicle sizes (H5). All in all, H5 was only confirmed for the perceived safety.

## Discussion

This study investigated the interplay of vehicle kinematics and eHMI for the interaction between pedestrians and two differently sized AVs in a shared space as an example of a low-speed and low-distance traffic scenario. The results indicated that pedestrians' willingness to cross is influenced by the size of the AV. Moreover, the use of vehicle kinematics and eHMI communication can lead to high willingness to cross, and high trust- and safety ratings. Nevertheless, when the dynamic eHMI indicated a yielding intent by the vehicle, although the vehicle did not yield, pedestrians' perceived safety still increased compared to when no contradictory explicit information was given by the eHMI.

### AV's Joint Communication via Vehicle Kinematics and eHMI

Previous research showed that implicit communication signals, i.e., vehicle kinematics, helped pedestrians to decide whether to cross a street or not (Dey and Terken, 2017; Lee et al., 2020). This was also found to be true for pedestrians' interaction with AVs (Risto et al., 2017; Dey et al., 2020a). The results of this study also supported the high relevance of vehicle kinematics for pedestrians' interactions with two differently sized AVs in a shared space. Pedestrians focused on what the AV implicitly communicated and were more willing to cross, indicated a higher trust, and felt safer when a yielding intent was communicated implicitly by the AV. However, it needs to be addressed that the AVs decelerated in two steps in the yielding conditions. Therefore, no clear assumption can be made about how exactly each deceleration might have influenced pedestrians' willingness, trust, and perceived safety and when exactly. Nevertheless, the two-step deceleration presents a realistic motion pattern for AVs as in shared spaces multiple TPs will interact together and, thus, AVs will need to adapt to their surrounding dynamically. This should be further investigated in more complex traffic scenarios including more than just one pedestrian. Regarding explicit communication, previous research manifested that additional explicit communication can be beneficial in low-speed and low-distance traffic areas to clarify misunderstandings before they could actually result in accidents (Färber, 2016; Habibovic et al., 2018; Lee et al., 2020). In this study, both AVs were perceived as more trustworthy and safer when they were equipped with a dynamic eHMI compared to a static eHMI or no eHMI at all. Furthermore, the participants indicated a higher willingness to cross when a dynamic eHMI was presented which is consistent with previous findings (Clercq et al., 2019; Dey et al., 2019; Schieben et al., 2019a,b; Ackermans et al., 2020; Kaleefathullah et al., 2020; Lau et al., 2021a). Nevertheless, it needs to be pointed out that the online-based experimental approach and the experimental setting could have had an effect on pedestrians' subjective evaluation which needs to be addressed in future studies for further interpretation and comparison (Fuest et al., 2020).

Regarding the interplay of vehicle kinematics and eHMI, pedestrians' perceived safety increased with an explicitly communicating dynamic eHMI in combination with an implicitly communicated yielding intent (kinematics). Additionally, the support of the vehicle kinematics and the dynamic eHMI was perceived as rather high for pedestrians' willingness to cross when the vehicle yielded. This stands in line with previous studies showing that a yielding intent that is communicated implicitly and explicitly can support the interaction between pedestrians and AVs (Dey et al., 2020a; Rettenmaier and Bengler, 2021). Surprisingly, the dynamic eHMI still increased pedestrians' perceived safety when the vehicle did not yield. In this non-matching condition, the dynamic eHMI falsely communicated a yielding intent although the vehicle did not yield. Additionally, when the AV did not yield but the dynamic eHMI indicated so, the participants seem to overestimate the support of the vehicle kinematics and the dynamic eHMI. Overall, these findings indicated possible negative effects of eHMIs which were described in previous studies (Kitazaki and Daimon, 2018; Holländer et al., 2019; Lee et al., 2021). However, these results stand in clear contrast to a study by Dey et al. (2020a) that showed that pedestrians tended to rely on the implicit communication signals, i.e., the vehicle braking behavior when the eHMI signal contradicted the vehicle behavior. In this study, the pedestrians did not elaborate on the presented information signals correctly but rather shifted their attention to salient explicit communication signals to make assumptions about the vehicle's intention (Moussaïd et al., 2011). According to Petty and Cacioppo (1986), one would assume that pedestrians tended to elaborate on the presented information via the peripheral route in this study. In urban traffic, pedestrians' over-trust in eHMIs would present a high-risk traffic scenario and could have safety-critical consequences for pedestrians.

In conclusion, the results highlighted the importance of the interplay of vehicle kinematics and eHMI. If the information by vehicle kinematics and eHMI are well-coordinated, the combination showed a great potential to positively influence pedestrians' interaction with differently sized AVs. Nonetheless, if both communication means were not well-coordinated, negative effects did occur, i.e., pedestrians' safety was influenced by the dynamic eHMI even though it transmitted contradictory signals to the vehicle kinematics. Therefore, this study demonstrated the importance of a well-coordinated holistic communication approach to enable a safe and efficient interaction between pedestrians and AVs.

### Effect of Vehicle Size

This experimental online study compared two differently sized AVs, a smaller AV, and a larger AV. The results supported previous assumptions about the effect of vehicle size, i.e., a larger AV was perceived as significantly more threatening, more dangerous, less pleasant, stronger, and closer compared to the smaller AV in the videos. Furthermore, pedestrians indicated a higher willingness to cross for a smaller AV compared to a larger AV. However, no effect of the vehicle size was found on pedestrians' trust and perceived safety. This stands in contrast to previous findings by Lau et al. (2021a) who showed that a smaller AV was perceived as safer and affectively more positive compared to a larger AV. Nevertheless, this study's video sequences presented only short interactions and, thus, a realistic interaction could have been limited. However, the idea was to create short and uncertain situations in which the pedestrians should make a fast and intuitive decision. This was done with the overall goal to get insights into pedestrians' subjective experiences.

For both AVs, the combination of dynamic eHMI and an implicit yielding intent via the vehicle kinematics supported pedestrians' willingness to cross, trust, and perceived safety in this study. Nevertheless, when the dynamic eHMI showed contradictory signals to the vehicle kinematics, pedestrians became equally indecisive for both AVs although pedestrians perceived higher risk by the larger AV (according to the data validation check). As previously mentioned, this finding showed that pedestrians might have resorted to salient explicit communication signals even for their interaction with a larger-sized AV. Previous studies that revealed possible negative effects of eHMI communication, i.e., over-trust, primarily focused on pedestrians' interaction with a smaller AV (Holländer et al., 2019; Lee et al., 2021). This study also illustrated possible negative effects of eHMIs on pedestrians' interaction with a larger AV. If pedestrians would have initiated a crossing under these conditions in real urban traffic, their interaction with AVs could have had fatal consequences.

All in all, the results supported the assumption that the effect of size could also influence the future interaction between pedestrians and differently sized AVs. When the dynamic eHMI was presented in line with a yielding behavior, eHMIs supported the interaction with both AVs. However, when the dynamic eHMI contradicted the vehicle kinematics, pedestrians' perceived safety was influenced in a negative manner. In conclusion, this study could contribute to further research on the effect of vehicle size focusing on pedestrians' interaction with AVs in terms of subjective measurements and the investigation of a shared space.

### Limitations

This study was an experimental online study in which the participants took part from their private computers. Therefore, pedestrians' perception of the vehicle sizes and the perception of the driving behavior could be limited due to the experimental setting (Petzoldt et al., 2018; Fuest et al., 2020). If the perception is limited, a greater focus can possibly be placed on the visually present stimulus, i.e., the eHMI. Thus, further investigation of the parameter (vehicle size, vehicle kinematics) in a more ecologically valid environment is required. Although we provided detailed guidelines for the participation in this study, the experimental setting could not be fully controlled, e.g., light sources or the monitor size. Thus, the internal validity might also have been limited due to the experimental setting. Moreover, the participants were not able to ask further questions during the experiment. Nevertheless, a major focus was placed on the video functionality and a manipulation check and a data validation check were conducted before and after the experiment. Future studies should be conducted in-person and under more controlled experimental conditions to avoid any influencing factors by the testing environment. Furthermore, the participants rated all dependent variables (willingness to cross, trust, perceived safety) after they saw the videos. Therefore, changes in the subjective evaluation during the video presentation could not be addressed and no specific determination time points can be identified for the subjective measures. This study focused primarily on subjective measurements to investigate pedestrians' interaction with differently sized AVs. However, it needs to be addressed how pedestrians' crossing behavior could be influenced by, e.g., the vehicle size, by focusing on objective measures (Petzoldt, 2016; Beggiato et al., 2017; Ackermann et al., 2019b; Hensch et al., 2021).

### Future Work

Future work will focus on the investigation of the interplay of vehicle kinematics and eHMI in more ecologically valid experimental settings, e.g., virtual-reality environments or real traffic. The overall goal is to enable the participants a realistic interaction with the differently sized AVs. Moreover, future studies will focus on possible cultural differences in the communication with AVs in general and how culture might affect the interaction with differently sized AVs presenting implicit and explicit communication signals with a larger sample size (Färber, 2016; Weber et al., 2019). Additional future work should focus on more complex environments, i.e., more than only one pedestrian interacting with one AV, and on the consideration of different age groups, e.g., older pedestrians. Furthermore, the vehicle kinematics were only manipulated in two stages (yielding, and non-yielding). In future studies, the vehicle kinematics should be varied in more stages in combination with an eHMI and with a focus on differently sized vehicles Additionally, future studies should focus on the continuous recording of pedestrians' willingness to cross, trust, and perceived safety to be able to put the subjective measurements in relation to the vehicle kinematics during the vehicle's approach. Furthermore, qualitative feedback from the participants at the end of the experiment could also help to receive further insights into participants' experiences and should be included in future studies.

## Conclusion

This study investigated pedestrians' interaction with two differently sized AVs in a shared space as an example of a traffic scenario of low-speed and low-distance. Current research lacks not only standardized requirements of eHMIs for AVs but also, official requirements for a holistic communication approach, i.e., the combination of vehicle kinematics and eHMI for differently sized AVs. This study underlined the great potential of a holistic communication approach when both communication tools are well-coordinated. Nevertheless, the findings also highlighted possible negative effects of eHMIs when they were not coordinated correctly, i.e., when the eHMI message contradicted the vehicle kinematics. The consequences are fatal and would even be more serious for pedestrians' interaction with larger-sized AVs. This study's results showed that a holistic communication strategy that consisted of well-coordinated implicit and explicit communication signals by the vehicle kinematics and the eHMI contributed to a well-working interaction. However, the major focus should be put on the precise coordination of eHMI and vehicle kinematics as the participants tended to focus on explicit communication signals even though they were contradictory to the vehicle kinematics in this study. A well-coordinated holistic communication strategy will set the standard on how pedestrians will safely interact with differently sized AVs in future urban traffic.

## Data Availability Statement

The raw data supporting the conclusions of this article will be made available by the authors, without undue reservation.

## Ethics Statement

The studies involving human participants were reviewed and approved by Ethics boards of the German Aerospace Center (DLR), Cologne, Germany. The patients/participants provided their written informed consent to participate in this study. Written informed consent was obtained from the individual(s) for the publication of any potentially identifiable images or data included in this article.

## Author Contributions

ML: conceptualization, methodology, data curation, writing—original draft, visualization, validation, resources, formal analysis, investigation, software, writing—review and editing, and project administration. MJ: writing—review and editing and supervision. MO: conceptualization, methodology, visualization, validation, writing—review and editing, resources, supervision, project administration, and funding acquisition. All authors contributed to the article and approved the submitted version.

## Funding

This research was conducted within the project CADJapanGermany: HF which was funded by the Federal Ministry of Education and Research of Germany.

## Conflict of Interest

The authors declare that the research was conducted in the absence of any commercial or financial relationships that could be construed as a potential conflict of interest.

## Publisher's Note

All claims expressed in this article are solely those of the authors and do not necessarily represent those of their affiliated organizations, or those of the publisher, the editors and the reviewers. Any product that may be evaluated in this article, or claim that may be made by its manufacturer, is not guaranteed or endorsed by the publisher.

## References

[B1] Ackermann C. Beggiato M. Bluhm L.-F. Löw A. Krems J. F. (2019b). Deceleration parameters and their applicability as informal communication signal between pedestrians and automated vehicles. Transp. Res. Part F Traffic Psychol. Behav. 62, 757–768. 10.1016/j.trf.2019.03.006

[B2] Ackermann C. Beggiato M. Schubert S. Krems J. F. (2019a). An experimental study to investigate design and assessment criteria: what is important for communication between pedestrians and automated vehicles? Appl. Ergon. 75, 272–282. 10.1016/j.apergo.2018.11.002 30509537

[B3] Ackermans S. Dey D. Ruijten P. Cuijpers R. H. Pfleging B. (2020). The effects of explicit intention communication, conspicuous sensors, and pedestrian attitude in interactions with automated vehicles, in Proceedings of the 2020 CHI Conference on Human Factors in Computing Systems, eds Bernhaupt R. Mueller F. Verweij D. Andres J. McGrenere J. Cockburn A.. (New York, NY: ACM), 1–14. 10.1145/3313831.3376197

[B4] Beggiato M. Witzlack C. Krems J. F. (2017). Gap acceptance and time-to-arrival estimates as basis for informal communication between pedestrians and vehicles, in Proceedings of the 9th International Conference on Automotive User Interfaces and Interactive Vehicular Applications, eds Löcken A. Boll S. Politis I. Osswald S. Schroeter R. Large D.. (New York, NY: ACM), 50–57. 10.1145/3122986.3122995

[B5] Bengler K. Rettenmaier M. Fritz N. Feierle A. (2020). From HMI to HMIs: Towards an HMI Framework for Automated Driving. Information 11, 61. 10.3390/info11020061

[B6] Böckle M.-P. Brenden A. P. Klingegård M. Habibovic A. Bout M. (2017). SAV2P – Exploring the impact of an interface for shared automated vehicles on pedestrians' experience, in Proceedings of the 9th ACM Conference on Automotive User Interfaces and Interactive Vehicular Applications (AutomotiveUI'17), eds Boll S. Pfleging B. Donmez B. Politis I. Large D. (New York, NY: ACM), 136–140. 10.1145/3131726.3131765

[B7] Caird J. K. Hancock P. A. (1994). The perception of arrival time for different oncoming vehicles at an intersection. Ecol. Psychol. 6, 83–109. 10.1207/s15326969eco0602_1

[B8] Clamann M. Aubert M. Cummings M. L. (2017). Evaluation of vehicle-to-pedestrian communication displays for autonomous vehicles, in 96th Annual Research Board Meeting (Washington, DC: Transport Research Board), 6–12.

[B9] Clercq K. de Dietrich A. Nuñez Velasco J. P. Winter J. de Happee R. (2019). External human-machine interfaces on automated vehicles: effects on pedestrian crossing decisions. Hum. Factors 61, 1353–1370. 10.1177/0018720819836343 30912985 PMC6820125

[B10] Cohen J. (1988). Statistical Power Analysis for the Behavioral Sciences, 2nd Edn. Hillsdale, NJ: L. Erlbaum Associates.

[B11] Deb S. Hudson C. R. Carruth D. W. Frey D. (2018). Pedestrians receptivity in autonomous vehicles: exploring a video-based assessment, in Proceedings of Human Factors and Ergonomics Society Annual Meeting, Vol. 6 (Philadelphia, PA: Human Factors and Ergonomics Society), 2061–2065. 10.1177/1541931218621465

[B12] DeLucia P. R. (2008). Critical roles for distance, task, and motion in space perception: initial conceptual framework and practical implications. Hum. Factors 50, 811–820. 10.1518/001872008X312297 19110841

[B13] DeLucia P. R. (2013). Effects of size on collision perception and implications for perceptual theory and transportation safety. Curr. Dir. Psychol. Sci. 22, 199–204. 10.1177/0963721412471679

[B14] Dey D. Ackermans S. Martens M. Pfleging B. Terken J. (2022). Interactions of automated vehicles with road users, in Studies in Computational Intelligence. User Experience Design in the Era of Automated Driving, Vol. 980, eds Riener A. Jeon M. Alvarez I. (Cham: Springer International Publishing), 533–581. 10.1007/978-3-030-77726-5_20

[B15] Dey D. Habibovic A. Löcken A. Wintersberger P. Pfleging B. Riener A.. (2020b). Taming the eHMI jungle: a classification taxonomy to guide, compare, and assess the design principles of automated vehicles' external human-machine interfaces. Transp. Res. Interdiscipl. Perspect. 7, 100–174. 10.1016/j.trip.2020.100174

[B16] Dey D. Martens M. Eggen B. Terken J. (2017). The impact of vehicle appearance and vehicle behavior on pedestrian interaction with autonomous vehicles, in AutomotiveUI '17: Proceedings of the 9th International Conference on Automotive User Interfaces and Interactive Vehicular Applications Adjunct, eds Löcken >A. Boll >S. Politis >I. Osswald >S. Schroeter >R. Large >D.. (New York, NY: ACM), 158–162. 10.1145/3131726.3131750

[B17] Dey D. Martens M. Eggen B. Terken J. (2019). Pedestrian road-crossing willingness as a function of vehicle automation, external appearance, and driving behaviour. Transport. Res. Part F Traffic Psychol. Behav. 65, 191–205. 10.1016/j.trf.2019.07.027

[B18] Dey D. Matviienko A. Berger M. Pfleging B. Martens M. Terken J. (2020a). Communicating the intention of an automated vehicle to pedestrians: the contributions of eHMI and vehicle behavior. IT Inform. Technol. 63, 123–141. 10.1515/itit-2020-0025

[B19] Dey D. Terken J. (2017). Pedestrian interaction with vehicles: roles of explicit and implicit communication, in Proceedings of the 9th International Conference on Automotive User Interfaces and Interactive Vehicular Applications, eds Löcken A. Boll S. Politis I. Osswald S. Schroeter R. Large D.. (New York, NY: ACM), 109–113. 10.1145/3122986.3123009

[B20] Dietrich A. Maruhn P. Schwarze L. Bengler K. (2020). Implicit communication of automated vehicles in urban scenarios: effects of pitch and deceleration on pedestrian crossing behavior, in Advances in Intelligent Systems and Computing. Human Systems Engineering and Design II, Vol. 1026, eds Ahram T. Karwowski W. Pickl S. Taiar R. (Cham: Springer International Publishing), 176–181. 10.1007/978-3-030-27928-8_27

[B21] Ezzati Amini R. Katrakazas C. Antoniou C. (2019). Negotiation and decision-making for a pedestrian roadway crossing: a literature review. Sustainability 11, 6713. 10.3390/su11236713

[B22] Faas S. M. Mathis L.-A. Baumann M. (2020). External HMI for self-driving vehicles: which information shall be displayed? Transport. Res. Part F Traffic Psychol. Behav. 68, 171–186. 10.1016/j.trf.2019.12.009

[B23] Faas S. M. Stange V. Baumann M. (2021). Self-driving vehicles and pedestrian interaction: does an external human-machine interface mitigate the threat of a tinted windshield or a distracted driver? Int. J. Hum. Comput. Interact. 37, 1364–1374. 10.1080/10447318.2021.1886483

[B24] Färber B. (2016). Communication and communication problems between autonomous vehicles and human drivers. in Autonomous Driving, eds Maurer M. Gerdes J. C. Lenz B. Winner H. (Berlin: Springer), 125–144. 10.1007/978-3-662-48847-8_7

[B25] Field A. (2009). Discovering Statistics using SPSS. London; Los Angeles, CA: Sage Publications.

[B26] Franke T. Attig C. Wessel D. (2018). A personal resource for technology interaction: development and validation of the affinity for technology interaction (ATI) scale. Int. J. Hum.Comput. Interact. 35, 456–467. 10.1080/10447318.2018.1456150

[B27] Fuest T. Schmidt E. Bengler K. (2020). Comparison of methods to evaluate the influence of an automated vehicle's driving behavior on pedestrians: wizard of Oz, virtual reality, and video. Information 11, 291. 10.3390/info11060291

[B28] German Road Traffic Regulations StVO. (2013). (Federal Law Gazette I, pp. 367), last amended by Article 13 of the Act of 12 July 2021 (Federal Law Gazette I, p. 3091).

[B29] Habibovic A. Lundgren V. M. Andersson J. Klingegård M. Lagström T. Sirkka A.. (2018). Communicating intent of automated vehicles to pedestrians. Front. Psychol. 9, 1336. 10.3389/fpsyg.2018.01336 30131737 PMC6090516

[B30] Hensch A.-C. Beggiato M. Schömann M. X. Krems J. F. (2021). Different types, different speeds – the effect of interaction partners and encountering speeds at intersections on drivers' gap acceptance as an implicit communication signal in automated driving, in Lecture Notes in Computer Science. HCI in Mobility, Transport, and Automotive Systems, Vol. 12791, ed Krömker H. (Cham: Springer International Publishing), 517–528. 10.1007/978-3-030-78358-7_36

[B31] Holländer K. Wintersberger P. Butz A. (2019). Overtrust in external cues of automated vehicles. in Proceedings of the 11th International Conference on Automotive User Interfaces and Interactive Vehicular Applications (New York, NY: ACM), 211–221. 10.1145/3342197.3344528

[B32] Horswill M. S. Helman S. Ardiles P. Wann J. P. (2005). Motorcycle accident risk could be inflated by a time to arrival illusion. Optometry 82, 740–746. 10.1097/01.opx.0000175563.21423.50 16127340

[B33] Kaleefathullah A. A. Merat N. Lee Y. M. Eisma Y. B. Madigan R. Garcia J.. (2020). External human–machine interfaces can be misleading: an examination of trust development and misuse in a CAVE-based pedestrian simulation environment. Hum. Factors. 10.1177/0018720820970751 33242999 PMC9421345

[B34] Kettwich C. Dodiya J. Wilbrink M. Schieben A. M. (2019). Light-based communication of automated vehicles with other traffic participants - a usability study in a Virtual Reality environment, in Darmstädter Lichttechnik: Vol. 18. Proceedings of the 13th International Symposium on Automotive Lightning, ed Khanh T. Q. (Munich: utzverlag GmbH).

[B35] Kitazaki S. Daimon T. (2018). To make automated vehicles communicative and sociable on roads, in Proceedings of the ITS World Congress (Kopenhagen, Denmark).

[B36] Lau M. Jipp M. Oehl M. (2021b). Investigating the interplay between eHMI and dHMI for automated buses: How do contradictory signals influence a pedestrian's willingness to cross in 13th International Conference on Automotive 2021 (New York, NY: Association for Computing Machinery), 152–155. 10.1145/3473682.3480284

[B37] Lau M. Le D. H. Oehl M. (2021a). Design of external human-machine interfaces for different automated vehicle types for the interaction with pedestrians on a shared space, in Lecture Notes in Networks and Systems. Proceedings of the 21st Congress of the International Ergonomics Association (IEA 2021), Vol. 221, eds Black N. L. Neumann W. P. Noy I. (Cham: Springer International Publishing), 710–717. 10.1007/978-3-030-74608-7_87

[B38] Lee J. Daimon T. Kitazaki S. (2021). Negative effect of external human-machine interfaces in automated vehicles on pedestrian crossing behaviour: a virtual reality experiment, in Lecture Notes in Networks and Systems. Proceedings of the 21st Congress of the International Ergonomics Association (IEA 2021), Vol. 221, eds Black N. L. Neumann W. P. Noy I. (Cham: Springer International Publishing), 718–725. 10.1007/978-3-030-74608-7_88

[B39] Lee Y. M. Madigan R. Giles O. Garach-Morcillo L. Markkula G. Fox C.. (2020). Road users rarely use explicit communication when interacting in today's traffic: implications for automated vehicles. Cogn. Technol. Work 5, 145. 10.1007/s10111-020-00635-y

[B40] Lee Y. M. Madigan R. Markkula G. Pekkanen J. Merat N. Avsar H.. (2019). InterACT Deliverables 6.1. Methodologies for the Evaluation and Impact Assessment of the interACT Solutions. Available online at: https://www.interact-roadautomation.eu/wp-content/uploads/interACT_D6.1_01082019_v1.0_uploadWebsite_approved_reduced-size-1.pdf (accessed January 11, 2022).

[B41] Lee Y. M. Madigan R. Uzondu C. Garcia J. Romano R. Markkula G.. (2022). Learning to interpret novel eHMI: the effect of vehicle kinematics and eHMI familiarity on pedestrians' crossing behavior. J. Safe Res. 80, 270–280. 10.31234/osf.io/2xub4 35249607

[B42] Leiner D. (2019). SoSci Survey (Version 3.1.06) [Computer software]. Available online at: https://www.soscisurvey.de (accessed October 07, 2021).

[B43] Levulis S. J. DeLucia P. R. Yang J. Nelson V. (2018). Does perceived harm underlie effects of vehicle size on overtaking judgments during driving? Proc. Hum. Factors Ergon. Soc. Annu. Meet. 62, 1384–1388. 10.1177/1541931218621316

[B44] Li Y. Cheng H. Zeng Z. Liu H. Sester M. (2021). Autonomous vehicles drive into shared spaces: eHMI design concept focusing on vulnerable road users, in Proceedings of 2021 IEEE 24th International Conference on Intelligent Transportation Systems (Indianapolis, IN: IEEE), 1729–1736. 10.1109/ITSC48978.2021.9564515

[B45] Lundgren V. M. Habibovic A. Andersson J. Lagström T. Nilsson M. Sirkka A.. (2017). Will there be new communication needs when introducing automated vehicles to the urban context? in Advances in Human Aspects of Transportation. Advances in Intelligent Systems and Computing, eds Stanton N. Landry S. Di Bucchianico G. Vallicelli A. (Cham: Springer), 485–497. 10.1007/978-3-319-41682-3_41

[B46] Mahadevan K. Somanath S. Sharlin E. (2018). Communicating awareness and intent in autonomous vehicle-pedestrian interaction, in Proceedings of the 2018 CHI Conference on Human Factors in Computing Systems, (New York, NY: ACM), 1–12. 10.1145/3173574.3174003

[B47] Markkula G. Madigan R. Nathanael D. Portouli E. Lee Y. M. Dietrich A.. (2020). Defining interactions: a conceptual framework for understanding interactive behaviour in human and automated road traffic. Theor. Issues Ergon. Sci. 57, 1–24. 10.1080/1463922X.2020.1736686

[B48] Merat N. Louw T. Madigan R. Wilbrink M. Schieben A. (2018). What externally presented information do VRUs require when interacting with fully Automated Road Transport Systems in shared space? Accid. Anal. Prev. 118, 244–252. 10.1016/j.aap.2018.03.018 29615186

[B49] Moussaïd M. Helbing D. Theraulaz G. (2011). How simple rules determine pedestrian behavior and crowd disasters. Proc. Natl. Acad. Sci. U. S. A. 108, 6884–6888. 10.1073/pnas.1016507108 21502518 PMC3084058

[B50] Petty R. E. Cacioppo J. T. (1986). The elaboration likelihood model of persuasion, in Advances in Experimental Social Psychology, Vol. 19 (New York, NY: Academic Press. Inc), 123–205. 10.1016/S0065-2601(08)60214-216864311

[B51] Petzoldt T. (2016). Size speed bias or size arrival effect-How judgments of vehicles' approach speed and time to arrival are influenced by the vehicles' size. Accident Anal. Prev. 95(Pt A), 132–137. 10.1016/j.aap.2016.07.010 27428866

[B52] Petzoldt T. Ngoc Q. H.-S. Bogda K. (2017). Time to arrival estimates, (Pedestrian) gap acceptance and the size arrival effect, in Proceedings of the 9th International Driving Symposium on Human Factors in Driver Assessment, Training, and Vehicle Design: driving assessment 2017 (Iowa City, IA: University of Iowa), 44–50. 10.17077/drivingassessment.1613

[B53] Petzoldt T. Schleinitz K. Banse R. (2018). Potential safety effects of a frontal brake light for motor vehicles. IET Intell. Transport Syst. 12, 449–453. 10.1049/iet-its.2017.0321

[B54] Rasouli A. Kotseruba I. Tsotsos J. K. (2017). Agreeing to cross: how drivers and pedestrians communicate, in Proceedings of IEEE Intelligent Vehicles Symposium (IV) (Indianapolis, IN: IEEE), 264–269. 10.1109/IVS.2017.7995730

[B55] Rasouli A. Tsotsos J. K. (2020). Autonomous vehicles that interact with pedestrians: a survey of theory and practice. IEEE Trans. Intell. Transp. Syst. 21, 900–918. 10.1109/TITS.2019.2901817

[B56] Rettenmaier M. Albers D. Bengler K. (2020). After you?! – use of external human-machine interfaces in road bottleneck scenarios. Transp. Res. Part F Traffic Psychol. Behav. 70, 175–190. 10.1016/j.trf.2020.03.004

[B57] Rettenmaier M. Bengler K. (2021). The matter of how and when: comparing explicit and implicit communication strategies of automated vehicles in bottleneck scenarios. IEEE Open J. Intell. Transp. Syst. 2, 282–293. 10.1109/OJITS.2021.3107678

[B58] Risto M. Emmenegger C. Vinkhuyzen E. Cefkin M. Hollan J. (2017). Human-vehicle interfaces: the power of vehicle movement gestures in human road user coordination, in Proceedings of the 9th International Driving Symposium on Human Factors in Driver Assessment, Training, and Vehicle Design: driving assessment 2017 (Iowa City, IA: University of Iowa), 186–192. 10.17077/drivingassessment.1633

[B59] Schieben A. Wilbrink M. Dietrich A. Ruenz J. Portouli E. Amditis A.. (2020). Designing cooperative interaction of automated vehicles in mixed traffic environments: insights from the interACT project, in Proceedings of 8th Transport Research Arena TRA 2020, April 27-30 (Helsinki: Liikenne- ja viestintävirasto Traficom).

[B60] Schieben A. Wilbrink M. Kettwich C. Dodiya J. Oehl M. Weber F.. (2019b). "Testing external HMI designs for automated vehicles - an overview on user study results from the EU project interACT, in Presented at 19th Conference on Automated Driving (Munich: Tagung Automatisiertes Fahren), 9.

[B61] Schieben A. Wilbrink M. Kettwich C. Madigan R. Louw T. Merat N. (2019a). Designing the interaction of automated vehicles with other traffic participants: design considerations based on human needs and expectations. Cogn. Technol. Work 21, 69–85. 10.1007/s10111-018-0521-z

[B62] Smeets J. B. J. Brenner E. Trébuchet S. Mestre D. R. (1996). Is judging time-to-contact based on tau? Perception 25, 583–590. 10.1068/p250583 8865299

[B63] Stanciu S. C. Eby D. W. Molnar L. J. St. Louis R. M. Zanier N. Kostyniuk L. P. (2018). Pedestrians/bicyclists and autonomous vehicles: how will they communicate? Transp. Res. Rec. 2672, 58–66. 10.1177/0361198118777091

[B64] Sucha M. Dostal D. Risser R. (2017). Pedestrian-driver communication and decision strategies at marked crossings. Accident Anal. Prev. 102, 41–50. 10.1016/j.aap.2017.02.018 28259827

[B65] Tyndall J. (2021). Pedestrian deaths and large vehicles. Econ. Transp. 26–27, 100219. 10.1016/j.ecotra.2021.100219

[B66] Weber F. Chadowitz R. Schmidt K. Messerschmidt J. Fuest T. (2019). Crossing the street across the globe: a study on the effects of eHMI on Pedestrians in the US, Germany and China, in Proceedings of HCI in Mobilitym Transport and Automotive Systems (Cham: Springer), 515–530. 10.1007/978-3-030-22666-4_37

[B67] Wickens C. (2021). Attention: theory, principles, models and applications. Int. J. Hum. Comput. Interact. 37, 403–417. 10.1080/10447318.2021.1874741

[B68] Wilbrink M. Lau M. Illgner J. Schieben A. Oehl M. (2021). Impact of external human–machine interface communication strategies of automated vehicles on Pedestrians' crossing decisions and behaviors in an urban environment. Sustainability 13, 8396. 10.3390/su13158396

[B69] World Health Organisation (2013). Global Status Report on Road 2013: supporting a decade of action.

